# Effects of a Supplement Containing a Cranberry Extract on Recurrent Urinary Tract Infections and Intestinal Microbiota: A Prospective, Uncontrolled Exploratory Study

**DOI:** 10.1089/jicm.2021.0300

**Published:** 2022-05-11

**Authors:** Michael Jeitler, Andreas Michalsen, Andreas Schwiertz, Christian S. Kessler, Daniela Koppold-Liebscher, Julia Grasme, Farid I. Kandil, Nico Steckhan

**Affiliations:** ^1^ Institute of Social Medicine, Epidemiology and Health Economics, Charité—Universitätsmedizin Berlin, corporate member of Freie Universität Berlin, and Humboldt-Universität zu Berlin, Berlin, Germany.; ^2^ Department of Internal and Integrative Medicine, Immanuel Hospital Berlin, Berlin, Germany.; ^3^ Institute of Microecology, Herborn, Germany.

**Keywords:** cranberry, urinary tract infection, complementary medicine, microbiome, integrative medicine, antibiotics

## Abstract

**Aim:**

Cranberries (*Vaccinium macrocarpon*) are traditionally used in prevention of urinary tract infections (UTIs). The authors' aim was to evaluate effects of a supplement containing cranberry extract, pumpkin seed extract, vitamin C, and vitamin B_2_ on recurrent uncomplicated UTIs in women and their intestinal microbiota.

**Methods:**

A prospective, uncontrolled exploratory study was conducted in women with recurrent uncomplicated UTIs. The primary exploratory outcome was the number of UTIs in a 6-month prospective observation period compared with a 6-month retrospective period. Further outcomes included number of antibiotics, quality of life (SF-36), intestinal microbiota (assessed by 16S rRNA amplicon sequencing), and evaluation questions. Parameters were assessed at baseline and after 1, 2, and 7 months (start of intake of cranberry supplement after 1 month for 6 months). *p*-Values were calculated with the pairwise Wilcoxon signed-rank test for α diversity and permutational multivariate analysis of variance.

**Results:**

Twenty-three women (aged 52.7 ± 12.4 years) were included in the study. Participants reported 2.2 ± 0.8 UTIs (at baseline) in the previous 6 months. After 6 months of cranberry intake, participants reported a significant decrease to 0.5 ± 0.9 UTIs (*p* < 0.001). Number of antibiotic therapies was also significantly (*p* < 0.001) reduced by 68% during 6 months of cranberry intake (0.14 ± 0.35) when compared with 6 months retrospectively (1.14 ± 0.71). The SF-36 physical component score increased from 44.9 ± 5.5 at baseline to 45.7 ± 4.6 at 7 months (*p* = 0.16). The SF-36 mental component score decreased slightly from the baseline value of 46.5 ± 6.5 to 46.2 ± 6.4 at 7 months (*p* = 0.74). No significant intragroup mean changes at genus, family, or species level for α and β diversity within the intestinal microbiota were found. In the evaluation questions, participants rated the cranberry extract positively and considered it beneficial. The supplement intake was safe.

**Conclusions:**

This study shows that women with recurrent uncomplicated UTIs benefit from cranberry intake. Future larger clinical studies with further investigation of the mechanisms of action are required to determine the effects of cranberries on participants with uncomplicated UTIs.

## Introduction

Recurrent, uncomplicated urinary tract infections (UTIs) are a frequent clinical problem not only in younger women but also in postmenopausal women with chronic diseases.^1^ Women have a 50% risk of UTIs over their lifetime and 20%–30% experience a subsequent UTI.^1^ Furthermore, UTIs represent a common and costly public health problem. The costs of managing acute UTIs range from $390 to $730 in the United States according to a recent analysis.^2^ The present recommendations for treatment of recurrent UTIs consist mainly of elevated intake of fluids, a combination of lifestyle measures, and the use of antibiotics.^1^ Unfortunately, antibiotic use exerts a negative impact on the gut microbiota, thereby possibly enhancing chronification of UTIs.^3^

Extracts from cranberries (*Vaccinium macrocarpon*) are traditionally used in the prevention of UTIs. Several randomized placebo-controlled trials demonstrated a preventive effect of cranberry extracts or cranberry juice on the rate of recurrent UTIs, for example, in younger women.^4^ However, a Cochrane meta-analysis from 2012 with 24 studies and over 4000 participants concluded that there is no clear evidence that cranberry supplementation may decrease the number of symptomatic UTIs for women with recurrent UTIs.^5^

Results of two more recent meta-analyses of 2017 indicated that cranberry extract reduced the risk of UTIs by 26%^6^ and 33%,^7^ respectively. A recent trial showed beneficial effects of cranberry juice in prevention of UTIs in younger women.^4^ Cranberry extracts can also reduce the administration of antibiotics, which is of utmost clinical relevance since the use of antibiotics can lead to emergence of antibiotic-resistant microorganisms.^7^

Cranberries have multiple biological properties, including effects on bacterial adhesion, biofilm formation, microbial growth, and immunomodulatory and anti-inflammatory activities.^8^ In particular, the cranberry oligosaccharides can contribute to many health benefits. As they can not be metabolized by the host, they are excreted in urine following consumption of cranberries and may thus contribute to the antiadhesive properties observed.^8^

Among the discussed mechanisms of action of cranberry extracts in UTIs, a putative antiadhesive effect against uropathogenic *Escherichia coli* (UPEC) is at the forefront. However, the primary mode of action and the compounds responsible for this effect are hitherto unknown. Flavones from cranberries may contribute to the antiadhesive activity.^9^ Recent metabolomic studies revealed that cranberry juice consumption significantly altered the urinary metabolome and increased urinary excretion of both exogenous and endogenous metabolites.^9^

Inhibition of adhesion of UPEC by cranberry extract and its metabolites was demonstrated in a recent randomized controlled trial.^10^ At least 36-mg cranberry proanthocyanidin (PAC) equivalents per day, divided into two doses (morning and evening), are required to mediate the observed antiadhesion bioactivity considered necessary to prevent bacterial adhesion to uroepithelial cells lining the bladder wall.^11^ Furthermore, it is most likely that polyphenols, flavonoids, and anthocyanins of cranberries have effects on the gut microbiota. In recent years, the involvement of intestinal microbiota has come into focus as a possible origin of recurrent UTIs.^8^

UPEC is responsible for the majority of UTIs (>65%).^1^ Its natural reservoir is the gut. From here, *Escherichia coli* can colonize the perineal area and the vagina and subsequently infect the urinary tract.^1^ Antibiotics have been widely used for the treatment of UTIs. However, due to the overuse of antibiotics, increasing resistance has been reported, thus reducing their efficacy and increasing the rate of recurrence.^1,12^ Furthermore, antibiotics have a detrimental impact on the gut microbiota, which has been shown to be an essential component of human health.^13,14^ It is known that a balanced intestinal microbiota provides colonization resistance to a number of pathogens.^15^

The aim of this study was to evaluate clinical effects of a cranberry supplement in women with recurrent uncomplicated UTIs and to assess changes in the intestinal microbiome.

## Methods

### Study design

Women with recurrent uncomplicated UTIs were enrolled in a prospective, uncontrolled exploratory study over 7 months. The study started with a baseline visit (V0), which included a retrospective history taking of the number of UTIs and of the number of antibiotic therapies in the previous 6 months. It was followed by a prospective 1-month run-in phase. Study visits took place after 1 (V1), 2 (V2), and 7 (V3) months (Table 1).

**Table 1.** Overview of the Study Parameters

Outcome	V0 (baseline)	V1 (1 month)	V2 (2 months)	V3 (7 months)
Number of UTIs	x	x	x	x
Number of antibiotics	x	x	x	x
SF-36 quality of life	x	x	x	x
Intestinal microbiota	x		x	x
Cranberry supplement (6 months)		x	x	x

Study visits at baseline (V0) and after 1 (V1), 2 (V2), and 7 (V3) months. Six-month intake of cranberry supplement from V1 to V3.

SF-36 quality of life, Medical Outcomes Study 36-Item Short-Form Questionnaire; UTIs, urinary tract infections.

The cranberry supplement was taken for 6 months from V1 to V3. At V0, V2, and V3, fecal samples were collected for the analysis of intestinal microbiota. The study protocol was approved by the ethics committee of the Charité—Universitätsmedizin Berlin, Berlin, Germany (EA1/268/16), and all participants gave their informed consent. Trial registration was done at clinicaltrials.gov (NCT03019874).

### Participants

Individuals with uncomplicated, chronic recurrent UTIs and aged 18 to 70 years were eligible to participate. Chronic recurrent urinary infections were defined as ≥3 infections per year or 2 infections in the last 6 months. Participants were excluded if they had renal insufficiency (glomerular filtration rate <60), any anatomical or known structural causes of UTIs, a regular consumption of probiotics or probiotic yogurt (at least 5 × per week), any intake of antibiotics in the past 4 weeks, any intake of phenprocoumon (or derivatives), or simultaneous participation in another study.

### Outcomes

#### Primary outcome

The primary outcome was the change in the number of UTIs after the 6-month intake of the cranberry supplement (total number of UTIs at V2 and V3) in comparison with the 6-month retrospective period assessed at V0. Therefore, UTIs were ascertained by a study physician at every study visit. Originally we planned the Acute Cystitis Symptom Score (ACSS) as the primary outcome. However, after inadequate adherence to this questionnaire, the clinical evaluation of UTIs by the physician was defined as the primary endpoint.

#### Secondary outcomes

Secondary outcomes included change in the number of antibiotic therapies following the 6-month consumption of cranberry supplements (total number of antibiotic therapies at V2 and V3) in comparison with the 6-month retrospective period assessed at V0. Antibiotics were ascertained by a study physician at every study visit. Quality of life was measured at V0, V1, V2, and V3 by the German version of the Medical Outcomes Study 36-Item Short-Form Questionnaire (SF-36).^16^

At V0, V2, and V3, fecal samples were collected. Analysis of the microbiota was performed by the Institute of Microecology (Herborn, Germany), as described elsewhere.^17^ Further analysis included responders (defined as no UTIs between V2 and V3 during cranberry intake) and self-reported compliance (defined as compliant to the recommended application—3 × 1 capsule daily).

#### Additional parameters

Moreover, expectation (at baseline) and evaluation questions (at V3) were asked using a numeric rating scale (1 = very to 5 = not at all).

### Cranberry supplement

The used cranberry product, named Cystorenal Cranberry plus, is a food supplement. The product has been notified to the Federal Office of Consumer Protection and Food Safety in accordance with the “§5 Food Supplements Ordinance” (German: Nahrungsergänzungsmittelverordnung). One capsule of Cystorenal Cranberry plus contains 300 mg of cranberry extract (including 12 mg of PACs), 100 mg of pumpkin seed extract, 20 mg of vitamin C, and 0.47 mg of riboflavin (vitamin B_2_). The recommended application is 3 × 1 capsule daily. The nutrients supplied in this dosage are in the nutritional–physiological range. The blend was composed in a holistic approach to maintain a healthy bladder.

In traditional medicine, cranberries are mainly used to prevent UTIs, while pumpkin seed preparations are used for the relief of lower urinary tract symptoms related to an overactive bladder.^18^ Vitamin C supports the maintenance of function of the immune system, and vitamin B_2_ is added to sustain the maintenance of healthy mucous membranes as occurring in the bladder. Cystorenal Cranberry plus contains no animal ingredients and is gluten and lactose free and free of preservatives. An high performance liquid chromatography chromatogram of the extract has been published before.^19^

### Statistical analyses

This study was conducted as an exploratory study. According to current knowledge, a biomathematical analysis of the microbiota using 16S sequencing can be expected to produce differential effects in *n* = 15–20 participants within a few weeks with large effect sizes. The sample size is based on the estimation of feasibility in accordance with the exploratory character of the observational study and aims at sufficiently supported generation of hypotheses for future studies.

All statistical analyses were done using the statistical programming language, R (version 3.5.2). The primary endpoint was calculated with the Wilcoxon signed-rank test. UTI frequencies were compared with Fisher's exact test for count data. The secondary endpoint (SF-36) was compared using the Friedman test, and *post hoc* analysis was done using the pairwise Wilcoxon signed-rank test.

Statistical analyses resulting in *p*-values were corrected using a false discovery rate correction for multiple testing. Statistical analysis was based on intention-to-treat analysis. Missing data were imputed with the Multivariate Imputation by Chained Equations package.

Sequence data were processed using Quantitative Insights Into Microbial Ecology (QIIME) 1.9.1.76. Quality cutoffs were performed using the QIIME standard at *Q* ≥ 25. Minimum and maximum sequence lengths with the QIIME default of 200 and 1000 bp were used. The sequences of four independent runs were merged in a single data set. Chimeras were removed using vsearch. Operational taxonomic units (OTUs) were chosen within 97% sequence identity. SILVA ribosomal RNA database release 128 was used to assign taxonomy and align sequences. Following removal of chloroplast and mitochondrial OTUs, further statistical analyses were done with R, version 3.5.4.

The vegan package, version 2.5, was used for rarefaction to even sequence depth and exclusion of taxa present in less than 10% of all samples. Alpha diversity indices for observed Chao1 and Shannon as well as β diversity indices such as Bray–Curtis were calculated with the phyloseq package (version 1.30). *p*-Values were calculated with the pairwise Wilcoxon signed-rank test for α diversity and permutational multivariate analysis of variance for β diversity with the package, vegan (dissimilarity index: Bray–Curtis), version 2.5.

Relative abundance values were transformed using the centered log-ratio transformation with the R package compositions. The authors then performed a statistical analysis of the differences in microbiota composition between time points for each taxon, by fitting linear mixed models with lmer, considering repeated sampling of individuals as random effects. *p*-Values were calculated using the difflsmeans function with the R package, lmertest.

## Results

Sixty-four women were screened for eligibility. Twenty-three women (aged 52.7 ± 12.4 years) were included in the study (Table 2). Participants were enrolled in March 2017; follow-ups were completed by February 2019. Overall, 23 participants completed V1 and V2, and V3 was completed by 20 participants. Twenty-three data sets were included in the final analysis.

**Table 2.** Baseline Characteristics

Variable	
*n*	23
Premenopausal, *n* (%)	10 (43.5)
Age, years	52.7 ± 12.4
BMI	23.5 ± 3.8
Systolic blood pressure	121.3 ± 13.4
Diastolic blood pressure	79.5 ± 7.4
Impairment of quality of life due to UTIs (NRS: 0 = not at all to 4 = very)	2.3 ± 1.1
UTIs in the past 6 months before enrollment	2.2 ± 0.8
SF-36 mental component	46.5 ± 6.5
SF-36 physical component	44.9 ± 5.5
Comorbidities, *n* (%)	
Hypothyroidism	4 (17.4)
Pollen allergy	3 (13)
Chronic back pain	2 (8.7)
Hypercholesterolemia	2 (8.7)
Atrial fibrillation	1 (4.3)
Chronic sinusitis	1 (4.3)
Gonarthrosis	1 (4.3)
Migraine	1 (4.3)
Diverticulosis	1 (4.3)

Mean ± standard deviation, unless otherwise specified.

BMI, body mass index; NRS, numeric rating scale; SF-36 quality of life, Medical Outcomes Study 36-Item Short-Form Questionnaire; UTIs, urinary tract infections.

Participants reported 2.2 ± 0.8 UTIs (at baseline) in the previous 6 months. Impairment of quality of life due to UTIs was moderate on average (Table 2). Expectations regarding cranberry intake, for example, on quality of life and reduction of impairment, were moderate to high on average (see Supplementary Table S1 for more information).

### Primary outcome

**FIG. 1.**
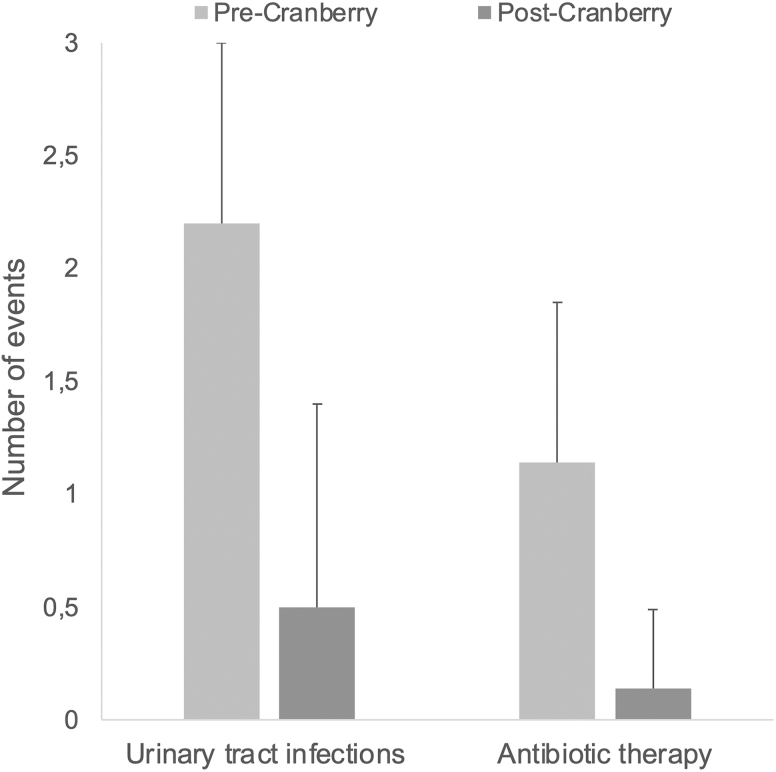
Number of UTIs (*left*) and number of antibiotic therapies (*right*). *Precranberry* treatment: participants reported (V0) 2.2 ± 0.8 (mean ± standard deviation) UTIs at baseline and 1.14 ± 0.71 antibiotic intake for the previous 6 months. *Postcranberry* treatment: at V3, after 6-month intake of the cranberry supplement, participants reported 0.5 ± 0.9 UTIs and 0.14 ± 0.35 antibiotic intake, both a significant decrease (*p* < 0.001). UTIs, urinary tract infections.

Participants reported 2.2 ± 0.8 UTIs (at baseline) for the previous 6 months. After 6 months of cranberry supplement intake, the participants reported 0.5 ± 0.9 UTIs, a significant decrease (*p* < 0.001) (Fig. 1).

### Secondary outcomes

The number of antibiotic therapies was significantly (*p* < 0.001) reduced after 6 months of cranberry supplement intake (0.14 ± 0.35 antibiotics) in comparison with the 6-month retrospective period assessed at baseline (1.14 ± 0.71 antibiotic intake). A reduction of 68% was achieved.

The SF-36 physical component score increased from V0 (44.9 ± 5.5) to V3 (45.7 ± 4.6) (*p* = 0.16). The SF-36 mental component score decreased slightly from V0 (46.5 ± 6.5) to V3 (46.2 ± 6.4) (*p* = 0.74). See Supplementary Table S2 and Supplementary Figure S1 for more information.

**FIG. 2.**
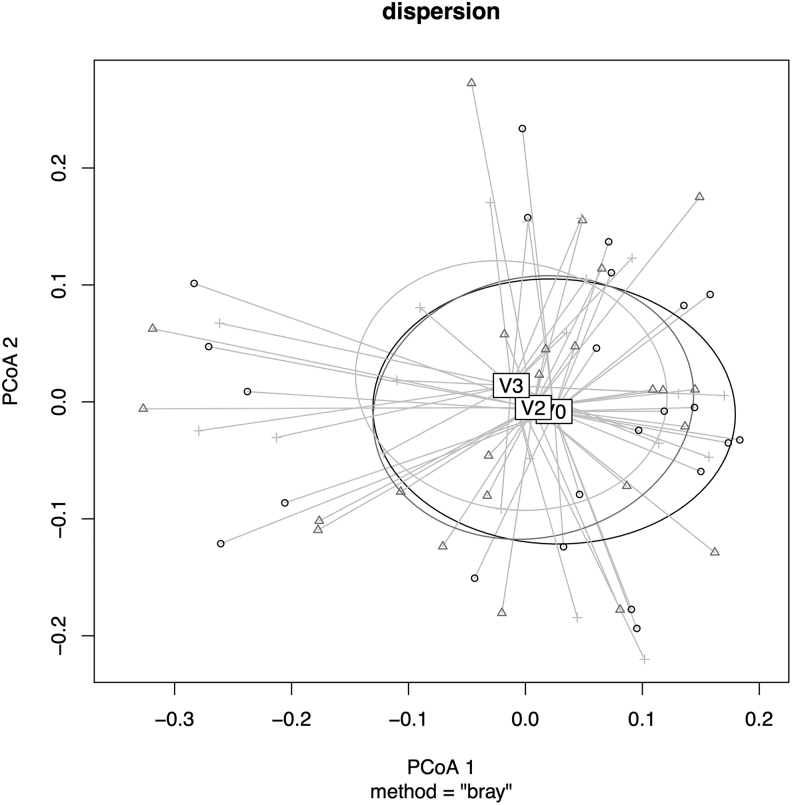
Analysis of multivariate homogeneity of group dispersion based on Bray–Curtis dissimilarity. Study visits at baseline (V0) and after 1 (V1), 2 (V2), and 7 (V3) months. Six-month intake of cranberry supplement from V1 to V3. PCoA, principal coordinate analysis axis.

No significant intragroup mean changes for α and β diversity in the intestinal microbiome were found when testing genus, family, or species level. Relative abundance values using the Bray–Curtis distance matrix were observed in the plot for the analysis of multivariate homogeneity of group dispersion. However, no clustering of time points was identified (Fig. 2). As depicted in Figure 2, Permutational multivariate analysis of variance results based on Bray–Curtis dissimilarities using relative abundance data were not significant (*F* = 0.36, *R*^2^ = 0.011, *p* = 0.99). Thus, because of high individual sample variance, a compositional change of the microbiota during the course of the interventions cannot be identified.

Relative relatedness of community members using (un-)weighted unifrac distances also revealed no effect on β diversity when only comparing repeated measures (see Supplementary Fig. S2 for more information). No change in the *Bacteroidetes–Firmicutes* ratio was observed (*p* = 0.075) (see Supplementary Fig. S3 for more information). However, *Firmicutes* became more dominant under cranberry intake in comparison with *Bacteroidetes*, although this trend is not significant.

Of the 23 women included, 13 participants (56.5%) were responders (defined as no UTIs between V1 and V3 during cranberry intake) and 14 participants (60.9%) were compliant (defined as compliant to the recommended application—3 × 1 capsule daily). To inspect compositional influences of compliant responders, an ordination plot is shown in Supplementary Figure S4 with more information. No unique direction seems to be dominating the star plot. Linear models of relative species abundance for both follow-up time points against the baseline are shown in Supplementary Table S3 (change between V0 and V2) and Supplementary Table S4 (change between V0 and V3).

None of the models indicated significant effects on repeated measures after adjustment. Analysis of responder versus nonresponder did not reveal any consistent change from baseline according to the classes. The variance reflects the intraindividual variance and does not represent a time effect on the microbiota (see Supplementary Fig. S5 for more information). A responder comparison on the phylum level revealed no significant compositional changes over time (see Supplementary Fig. S6 for more information).

### Evaluation questions

On average, participants rated the effects of cranberry intake as good and beneficial (Table 3).

**Table 3.** Evaluation Questions

Question	Mean ± SD
By consuming cranberries, my complaints have been significantly reduced	1.7 ± 0.9
Cranberry consumption has significantly improved my quality of life	1.9 ± 1
Cranberry consumption has reduced the intake of antibiotics	1.5 ± 0.8
I tolerated cranberry supplement well	1.1 ± 0.4
Participation in the study was worthwhile for me	1.4 ± 0.7
I can imagine continuing the consumption of cranberries	1.5 ± 1.0
I am satisfied with the result	1.6 ± 1

Numeric rating scale: 1 = very to 5 = not at all.

SD, standard deviation.

### Safety

Overall, cranberry intake was safe. Only mild, self-limiting adverse events occurred, which were not related to cranberry intake (*n* = 10 adverse events in *n* = 8 participants; assessed by the study physicians). One serious adverse event not related to cranberry intake occurred (traumatic brain injury with hospitalization).

## Discussion

This prospective, uncontrolled exploratory study was conducted to investigate potential effects of a supplement containing cranberry extract, pumpkin seed extract, and vitamins C and B_2_ on recurrent uncomplicated UTIs. It could be shown that the numbers of UTIs and antibiotics were reduced significantly. This is in accordance with previous studies on cranberries.^4,6,7^ However, the effect of cranberries on UTIs is not unambiguous.^5^ It can be speculated that the use of cranberries reduced the amount of pathogenic microbes in the urine. Recently, cranberry juice was reported to reduce UTIs in an animal model,^20^ and numerous *in vitro* experiments reported antimicrobial or antiadhesive properties of the phenolic components of cranberry products against *E. coli.*^21^

Interestingly, the authors were also able to show that the use of cranberry extract reduces the use of antibiotics. To date, a limited number of studies have examined the potential benefits of cranberry extract in treating symptoms of acute noncomplicated UTIs, or even whether cranberry extract might have a synergistic effect when combined with antibiotics.^22,23^

Few studies investigated the effects of cranberries on the microbiota using 16S rRNA sequencing, and it has been shown that cranberry consumption can modulate the gut microbiota.^24–26^ Rodriguez-Morato et al. showed a shift in the *Bacteroidetes*–*Firmicutes* ratio and an increase in commensals.^24^ In addition, cranberries attenuated the impact of the animal-based diet on microbiota composition, bile acids, and short-chain fatty acids.^24^

Moreover, cranberry consumption partially reversed the change of gut microbiota in colitic mice by increasing the abundance of potential beneficial bacteria (e.g., *Lactobacillus* and *Bifidobacterium)* and decreasing the abundance of potential harmful bacteria (e.g., *Sutterella* and *Bilophila).*^25^ Two studies used whole cranberry fruit powder made from freshly harvested cranberries,^24,25^ while a third used sweetened dried cranberries.^26^

Interestingly, the authors did not observe any changes within the microbiota. This may be due to the cranberry extract used in this study, which was a spray-dried juice extract standardized to PACs. Hence, the extract contained almost exclusively water-soluble components. Solid cell wall components were separated in advance and thus the fiber content of the extract was <1%. The remaining carbohydrates were primarily short-chain saccharides. The formerly seen effects of cranberries on the microbiota may thus have been due to the still incorporated high amounts of polysaccharides and oligosaccharides, which enabled microbial fermentation and growth.^24–26^

The link between the gut microbiota and urological health is of increasing clinical interest and has led to the concept of the female urinary microbiota.^27,28^ The microbiota of the urinary tract represent 21% of the known prokaryotic diversity associated with humans, and 62% of the organisms identified in urine can also be found in human intestinal microbiota.^29^ Although the authors were not able to find changes within the microbiota, a slight but not significant decrease of the *Bacteroidetes*–*Firmicutes* ratio was observed as shown in the study by Rodriguez-Morato et al. in a randomized, crossover, controlled feeding trial.^26^

This may be of interest as a dominance of species of the phylum *Firmicutes* has been indicated in women with no history of UTIs. It may be speculated that lactobacilli, which are members of the *Firmicutes* phylum and the major representatives of the healthy vaginal microbiota, play herein a role. It is known that lactobacilli colonize the vagina during puberty through the perianal route. Participants with recurrent UTIs had comparatively poor levels of lactobacilli.^30,31^

*E. coli*, a member of the *Enterobacteriaceae family*, is the major pathogen responsible for UTIs. O'Connor et al. were able to show that cranberry components modulate the microbiota by increasing the abundance of *Bacteroidaceae*, while decreasing the abundance of *Enterobacteriaceae.*^32^ It has been shown that PACs of cranberries inhibit *E. coli* adhesives (especially P-fimbria). The PACs from cranberries contain unusual double A-type linkages, which could be important structural features in the antiadhesion process.^33^ It has been shown that adhesion prevention is equally effective against both antibiotic-sensitive and antibiotic-resistant strains of *E. coli*^34^ and that adhesion prevention does not exert selective pressure that can lead to development of resistance.^35^

Dosages of at least 36 mg of PACs per day have been shown to have antiadhesive effects in urine. Increased dosages of 72 mg per day may provide an even longer protection.^11^ Kinetic data showed that the activity is highest at 36 and 72 mg after 6 h, but decreases significantly after 24 h, suggesting that it may be beneficial to consume more PACs than in this study.^11^ At 72 mg of PACs (two divided doses of 36 mg, morning and evening), the various metabolites of PACs (especially various anthocyanins) are still present and effective 24 h after cranberry consumption.^11^

However, the importance of the influence of PACs is controversial as PAC-free extracts may also exert antiadhesive effects,^36^ and in another study, no clinical effects were found at even higher PAC levels.^37^ The mechanism of action of cranberry extract is not yet fully understood. Presumably, synergistic effects, including the presence of soluble oligosaccharides of plant origin in urine, could be beneficial for urinary tract health through both prebiotic effects on the urinary microbiota and direct biological and chemical effects.^8^

A limitation of this study is the uncontrolled study design without placebo control, thus unspecific effects cannot be excluded. Furthermore, the small number of participants precludes a robust statistical analysis. However, this study was designed for exploratory purposes only. In addition, compliance was self-reported only. The overall compliance was moderate, which could be due to the frequent intake of 3 × 1 capsule daily.

Nevertheless, this study showed interesting results and as demonstrated for the intestinal microbiota,^38,39^ current evidence suggests that the urogenital microbiota play a major role in the occurrence and progression of UTIs.^40–42^ Hence, additional studies are needed to further clarify the complex host–microbiota interactions for progression of UTIs and the possible influence in relation to cranberry extracts.^43,44^

## Conclusions

In conclusion, this explorative study showed evidence that women with recurrent uncomplicated UTIs can benefit from cranberry intake regarding reduction of numbers of UTIs and antibiotic therapies. Cranberry intake was well accepted by the participants and considered safe. The authors could not observe any changes in α and β diversity of the intestinal microbiota.

To better evaluate the impact of cranberries on recurrent uncomplicated UTIs, well-designed double-blind trials are needed, which include longer-term follow-ups, larger sample sizes, and further study of the mechanisms of action. It is therefore necessary to further investigate the possible effects of cranberry oligosaccharides and other complex carbohydrates on the gut and urinary tract microbiota and their health benefits.

## Supplementary Material

Supplemental data

Supplemental data

Supplemental data

Supplemental data

Supplemental data

Supplemental data

Supplemental data

Supplemental data

Supplemental data

Supplemental data
