# Supplementary material for: Effects of a Supplement Containing a Cranberry Extract on Recurrent Urinary Tract Infections and Intestinal Microbiota: A Prospective, Uncontrolled Exploratory Study
Source: J Integr Complement Med. 2022 May 11;28(5):399–406. doi: 10.1089/jicm.2021.0300 (PMC9127832; doi:10.1089/jicm.2021.0300)
Supplement: Supplemental data [file Suppl_TableS1.docx]

Table 1: Expectation questions (NRS: 1-very to 5-not at all)

| **Question** | **mean±SD** |
| --- | --- |
| I expect a significant reduction in my impairment at the end of the study. | 1.6±0.6 |
| I expect difficulties in regularly taking the study drug. | 4.5±1 |
| I expect that I will have a better quality of life by participating in the study at the end of the study. | 1.8±0.9 |
| I expect to be able to participate more actively in everyday life again through the treatments. | 2.1±0.8 |
| I cannot imagine that participation in the study will change anything about my symptoms. | 4.1±0.9 |
| I expect that I will not suffer from recurring urinary tract infections afterwards. | 2.4±0.9 |
