# Supplementary material for: Effects of a Supplement Containing a Cranberry Extract on Recurrent Urinary Tract Infections and Intestinal Microbiota: A Prospective, Uncontrolled Exploratory Study
Source: J Integr Complement Med. 2022 May 11;28(5):399–406. doi: 10.1089/jicm.2021.0300 (PMC9127832; doi:10.1089/jicm.2021.0300)
Supplement: Supplemental data [file Suppl_TableS2.docx]

Table 2: Quality of life (SF-36) with physical and mental component scale during the study observation time

|  | **SF-36 physical component scale** | | | | **SF-36 mental component scale** | | | |
| --- | --- | --- | --- | --- | --- | --- | --- | --- |
|  | **V0** | **V1** | **V2** | **V3** | **V0** | **V1** | **V2** | **V3** |
| **M** | 44.9 | 45.0 | 44.2 | 45.7 | 46.5 | 44.6 | 45.7 | 46.2 |
| **SD** | 5.5 | 5.1 | 6.9 | 4.6 | 6.5 | 6.9 | 6.7 | 6.4 |
| **Min** | 33.03 | 36.73 | 26.12 | 33.63 | 28.26 | 23.79 | 25.91 | 25.86 |
| **Q1** | 41.75 | 41.00 | 40.69 | 44.09 | 44.64 | 43.21 | 44.07 | 45.13 |
| **Med** | 46.05 | 45.86 | 45.99 | 45.77 | 47.34 | 46.81 | 47.66 | 47.77 |
| **Q3** | 48.25 | 47.92 | 48.25 | 48.53 | 49.65 | 48.81 | 49.21 | 49.98 |
| **Max** | 56.08 | 56.08 | 57.15 | 55.87 | 57.37 | 51.18 | 54.98 | 52.46 |
| **MAD** | 2.27 | 3.18 | 3.65 | 1.98 | 2.32 | 2.77 | 2.19 | 2.21 |
| **IQR** | 15.23 | 11.19 | 22.13 | 14.91 | 21.40 | 25.02 | 23.30 | 24.12 |

V=visit, M=mean, SD=standard deviation, Min=minimum, Q1=first quartile, med=median, Q1=third quartile, Max=maximum, MAD=mean absolute deviation, IQR=interquartile range

Study visits at baseline (V0) and after 1 (V1), 2 (V2) and 7 (V3) months. 6-month intake of cranberry supplement from V1 to V3.
