# Supplementary material for: Effects of a Supplement Containing a Cranberry Extract on Recurrent Urinary Tract Infections and Intestinal Microbiota: A Prospective, Uncontrolled Exploratory Study
Source: J Integr Complement Med. 2022 May 11;28(5):399–406. doi: 10.1089/jicm.2021.0300 (PMC9127832; doi:10.1089/jicm.2021.0300)
Supplement: Supplemental data [file Suppl_FigS3.docx]

Fig. 3: *Bacteroidetes-Firmicutes* ratio over time using a non-parametric test and adjusted within-subjects comparisons

Study visits at baseline (V0) and after 1 (V1), 2 (V2) and 7 (V3) months. 6-month intake of cranberry supplement from V1 to V3.
