# Supplementary material for: Effects of a Supplement Containing a Cranberry Extract on Recurrent Urinary Tract Infections and Intestinal Microbiota: A Prospective, Uncontrolled Exploratory Study
Source: J Integr Complement Med. 2022 May 11;28(5):399–406. doi: 10.1089/jicm.2021.0300 (PMC9127832; doi:10.1089/jicm.2021.0300)
Supplement: Supplemental data [file Suppl_TableS3.docx]

Table 3: Linear models of relative species abundancies reflecting the change between V0-V2, ranked by fdr-adjusted p-values

| **Species** | **beta** | **se** | **p-value** |
| --- | --- | --- | --- |
| Collinsella aerofaciens | 0.7241881 | 0.2980273 | 0.6424096 |
| Bifidobacterium bifidum | -0.3956134 | 0.1767754 | 0.6424096 |
| Streptococcus peroris | 0.4373386 | 0.1969028 | 0.6424096 |
| Pseudobutyrivibrio ruminis | 0.1083427 | 0.0492686 | 0.6424096 |
| Parabacteroides distasonis | -0.8716761 | 0.4020579 | 0.6424096 |
