# Supplementary material for: Effects of a Supplement Containing a Cranberry Extract on Recurrent Urinary Tract Infections and Intestinal Microbiota: A Prospective, Uncontrolled Exploratory Study
Source: J Integr Complement Med. 2022 May 11;28(5):399–406. doi: 10.1089/jicm.2021.0300 (PMC9127832; doi:10.1089/jicm.2021.0300)
Supplement: Supplemental data [file Suppl_TableS4.docx]

Table 4: Linear models of relative species abundancies reflecting the change between V0-V3, ranked by fdr-adjusted p-values

| **Species** | **beta** | **se** | **p-value** |
| --- | --- | --- | --- |
| Rothia mucilaginosa | -0.2538727 | 0.1202447 | 0.7883454 |
| Propionibacterium freudenreichii | -0.2257239 | 0.1878137 | 0.7883454 |
| Bifidobacterium angulatum | -0.1323657 | 0.0800944 | 0.7883454 |
| Turicibacter sanguinis | -0.3502233 | 0.2947156 | 0.7883454 |
| Catenibacterium mitsuokai | -0.2978553 | 0.1834072 | 0.7883454 |
